# A Treatise on Pulmonary Consumption, &c.

**Published:** 1837-04

**Authors:** 


ART. XII.—*Traité de la Consomption Pulmonaire, &c.* Par JAMES CLARK, M.D. *Traduit de l'Anglais*, par HENRI LEBEAU, Medicin en chef de l'Hôpital Militaire de Bruxelles.—*Bruxelles*, 1836. 8vo. pp. 388.

*A Treatise on Pulmonary Consumption, &c.* By JAMES CLARK, M.D. &c.—*Brussels*, 1836.

THE above translation having come into our hands with other books from the Brussels press, we were curious to know how the translator had succeeded in rendering into his own language one of the most valuable of our practical works, and which we knew to be written in the accurate and chaste style which befits a scientific treatise. We are well aware how difficult it is to preserve, in a foreign idiom, the graces of style of the original language, and we are therefore content to forego this, which, after all, is a mere ornament, in translations; but we think it positively culpable in any one who is not sufficiently master of the language in which any work is written,—more particularly a scientific work,—to undertake a translation of it; because, in his hands, not merely the author's graces and niceties of diction disappear, but his opinions may be misrepresented, his statements altered, and his very facts falsified. And we are sorry to say, that all this has befallen Dr. Clark in the hands of Dr. Lebeau. It is evident that this gentleman has an imperfect knowledge of the English language; and indeed he seems, throughout his task, to have troubled himself very little with giving an adequate, or even an accurate, interpretation of the original. To say nothing of inaccuracies of slighter moment, which are numberless, the translator has in many cases quite misconceived his author's meaning; and in still more he has carelessly and loosely rendered passages in such a manner as to convey meanings considerably different from the author's. For instance: he makes him affirm and state many things as *positive* which, in the original, were given as merely *probable*, or perhaps as a mere *opinion:* whole lines are occasionally omitted; sentences, and even paragraphs, are formed, where none existed in the original, &c. &c.

We could substantiate these charges by examples from almost every page of Dr. Lebeau's volume; but, as this would not greatly interest our readers, we shall content ourselves with these general remarks, trusting that, should they reach the eye of the translator, he will, in preparing a new edition, (which, from the great merits of the treatise, it is likely soon to reach,) take care to remove blemishes which are no less injurious to his own reputation than to that of the original author. And we take this occasion of recommending to Dr. L., what we would earnestly recommend to all translators,—and certainly to our English translators, as much as to others,—viz. to submit his work to the examination of some literary friend, who is tolerably well acquainted with the language of the translation, but whose mother-tongue is the language of the original. By this means,—and we believe we may say by this means alone,—can foreign versions of scientific works be obtained, which can do justice to all parties. Had this canon been followed during the last few years in England, we should have been spared the necessity of animadverting, in terms of some severity, on several medical translations which have passed through our hands, since we commenced our labours in this Journal.

We are so persuaded of the intimate connexion between clear expression and clear thinking, and so conscious of the deficiency in literary skill of many who publish medical books, that we are determined to persevere in criticising the manner as well as the matter of the works that come before us. This observation applies with double force to *translations;* and we therefore give our young and industrious friends, who may be engaged in the very meritorious task of translating, this fair warning, that we will judge them rigidly when they come before us. That they may not, however, expect to find in us judges prejudiced against undertakings of this sort, we wish to say distinctly how much we approve of them, and shall lose no fair opportunity of promoting them: and, indeed, how much better would it be for their own reputation and for their profession, if many of our own young men, just entering on practice, would employ their leisure hours in translating some of the classic works of our continental brethren, instead of heaping up materials of future regret for themselves in the shape of new treatises; original, indeed, in form, but too often consisting of crude facts unsifted by experience, wild and illogical theories, or mere compilations of the matter without the spirit of books already well known and in everybody's hands? We take some credit to ourselves for having suggested tasks of this kind to some of our junior brethren; and we doubt not that they will hereafter have good reason for congratulating themselves that the medical literature of this country is indebted to them for some of its most valuable stores. The translations and translators of WALTHER and MÜLLER, and KRAMER and BOUILLAUD, will be remembered by British physicians and surgeons and physiologists, long after the majority of the "original works" of the present day are forgotten.

And here we cannot deny ourselves the pleasure of remarking, that we have been much gratified, in the prosecution of our labours concerning our own journal, to find how wide-spread is the knowledge of the chief continental languages among the junior members of the higher branches of the profession in this country. German, Italian, and French may be said to be now regarded as essential to the education of a physician. We are, at least, sure that we speak within bounds when we say that, for one member of the profession that was acquainted with these languages (more particularly the two first named) thirty years ago, there are now fifty. Other languages also we find are studied to an extent beyond what we had previously believed; for instance, Spanish and Portuguese, Dutch and Danish; and, as our readers will see by a notice in the present Number, even the tongues of the Sclavonian stem, Polish and Russian. It is needless to remark how beneficial an influence such an extended knowledge of the literature of medicine must eventually exert over its practice. It is at once a proof and a consequence of the great and general progress of medical science; but it is also, in a considerable degree, attributable to the general peace which has now so long and so happily prevailed, and which is daily uniting more and more closely into one brotherhood all the members of our common profession throughout the world. It will continue to be our pleasure and our pride to contribute, in any degree, to this most desirable result.

